# Clinical presentation and markers of cutaneous versus systemic mastocytosis: the mastocytosis center at brigham and women's hospital in Boston

**DOI:** 10.1186/1939-4551-8-S1-A141

**Published:** 2015-04-08

**Authors:** Kelly Yoshimi Kanamori, Pedro Giavina-Bianchi, Nathalia Pessoa Simis, Cem Akin, Mariana Castells

**Affiliations:** 1 University of São Paulo, Brazil; 2 Harvard Medical School, USA; 3 Brigham & Womens Hospital, USA

## Background

Mastocytosis is a group of heterogeneous diseases characterized by an abnormal expansion and accumulation of mast cells (MCs) in different tissues. While systemic mastocytosis (SM) is defined by the accumulation of MCs in bone marrow (BM) and different tissues, cutaneous mastocytosis (CM) is characterized as an accumulation of MC in the skin with no other organ involvement. How gender differences, tryptase levels and episodes of anaphylaxis affect cutaneous versus systemic mastocytosis is not well understood.

## Methods

We report a case series of 109 patients with SM and 59 with CM from the Mastocytosis Center at Brigham and Women’s Hospital in Boston. The diagnosis of SM and CM were done according to the International Classification of Mastocytosis from WHO. We reviewed gender, tryptase levels and episodes of anaphylaxis in both populations.

## Results

There was a female predominance which was more pronounced in SM (68,8%) than in CM (52,7%). The mean serum tryptase level was 11.5 ng/ml in CM, and 14.9% of CM patients had serum tryptase levels above 20 ng/ml. In SM patients the mean serum tryptase level was 97.2 ng/ml and 84% of the patients presented levels above 20 ng/ml. The symptoms of CM included pruritus, flushing, urticaria, and dermatographism and 10.8% of the patients (number) had episodes of anaphylaxis. The rate of anaphylaxis in SM patients was found to be 29.4% similar to the previously reported.

## Conclusions

A female predominance was seen in both cutaneous and systemic mastocytosis in contrast to other studies indicating that there is no gender predominance. Elevations of serum tryptase above 20 ng/ml and anaphylaxis were seen in a small proportion of patients with cutaneous mastocytosis, raising the question of the potential progression to systemic mastocytosis and the need to closely monitor this subset of patients.

